# High-Grade Osteosarcoma of the Foot: Presentation, Treatment, Prognostic Factors, and Outcome of 23 Cooperative Osteosarcoma Study Group COSS Patients

**DOI:** 10.1155/2018/1632978

**Published:** 2018-05-02

**Authors:** Anne J. Schuster, Leo Kager, Peter Reichardt, Daniel Baumhoer, Monika Csóka, Stefanie Hecker-Nolting, Susanna Lang, Sylvie Lorenzen, Regine Mayer-Steinacker, Thekla von Kalle, Matthias Kevric, Mathias Werner, Reinhard Windhager, Thomas Wirth, Stefan S. Bielack

**Affiliations:** ^1^Center for Pediatric, Adolescent and Women's Medicine, Olgahospital, Department of Pediatrics 5 (Oncology, Hematology, Immunology), Klinikum Stuttgart, Stuttgart, Germany; ^2^St. Anna Children's Hospital, Department of Paediatrics, Medical University of Vienna and Children's Cancer Research Institute (CCRI), Vienna, Austria; ^3^Department of Interdisciplinary Oncology, HELIOS Klinikum Berlin-Buch, Berlin, Germany; ^4^Bone Tumour Reference Centre (BTRC), Institute of Pathology, University Hospital of Basel and University of Basel, Basel, Switzerland; ^5^2nd Department of Pediatrics, Semmelweis University, Budapest, Hungary; ^6^Department of Pathology, Vienna General Hospital, Medical University of Vienna, Vienna, Austria; ^7^Department of Hematology and Oncology, Klinikum rechts der Isar Technische Universität München, Munich, Germany; ^8^Department of Internal Medicine III, University of Ulm, Ulm, Germany; ^9^Center for Pediatric, Adolescent and Women's Medicine, Olgahospital, Department of Pediatric Radiology, Klinikum Stuttgart, Stuttgart, Germany; ^10^Department of Orthopaedics, Medical University of Vienna, Vienna, Austria; ^11^Center for Pediatric, Adolescent and Women's Medicine, Olgahospital, Department of Pediatrics, Division of Pediatric Orthopedics, Klinikum Stuttgart, Stuttgart, Germany; ^12^Department of Pediatric Hematology and Oncology, University Children's Hospital Muenster, Muenster, Germany

## Abstract

Osteosarcoma of the foot is a very rare presentation of a rare tumor entity. In a retrospective analysis, we investigated tumor- and treatment-related variables and outcome of patients registered in the Cooperative Osteosarcoma Study Group (COSS) database between January 1980 and April 2016 who suffered from primary high-grade osteosarcoma of the foot. Among the 23 eligible patients, median age was 32 years (range: 6–58 years), 10 were female, and 13 were male. The tarsus was the most commonly affected site (*n*=16). Three patients had primary metastases. All patients were operated: 5 underwent primary surgery and 18 received surgery following preoperative chemotherapy. In 21 of the 23 patients, complete surgical remission was achieved. In 4 of 17 patients, a poor response to neoadjuvant chemotherapy was observed in the resected primary tumors. Median follow-up was 4.2 years (range: 0.4–18.5). At the last follow-up, 15 of the 23 patients were alive and 8 had died. Five-year overall and event-free survival estimates were 64% (standard error (SE) 12%) and 54% (SE 13%), which is similar to that observed for osteosarcoma in general. Event-free and overall survival correlated with primary metastatic status and completeness of surgery. Our findings show that high-grade osteosarcoma in the foot has a similar outcome as osteosarcoma of other sites.

## 1. Introduction

Bone tumors of the foot have been reported to be rare, and reported studies are limited to case reports and very few small cohort studies [1–6]. Of these tumors, 23–26% are malignant and only 4% represent osteosarcomas, whereas only 1% of all osteosarcomas occur in the foot [6–8].

To fill the current gap in literature, we evaluated in this study all patients with an osteosarcoma of the foot registered by the COSS to identify prognostic factors and to evaluate similarities and differences in outcome compared to other osteosarcoma sites.

## 2. Methods

### 2.1. Patient Eligibility

The analysis is based on all patients registered by the Cooperative German-Austrian-Swiss Osteosarcoma Study Group (COSS) since 1980 [9–15]. The study group's primary focus has generally been on patients with primary high-grade central osteosarcoma of bone under 40 years of age, but all other patients in a different age group or diagnosed with another type of osteosarcoma were also registered and followed.

All COSS studies were approved by the appropriate ethics and/or protocol review committee. Before registration and therapy, informed consent was obtained from all patients and/or their legal guardians, depending on patients' age.

This study is based on all patients with a primary, previously untreated high-grade osteosarcoma of the foot registered between January 1980 and April 2016 with a follow-up of at least 3 months.

### 2.2. Diagnostics

Diagnostic procedures used to define the extension of the primary tumor included conventional radiography in all studies, whereas computed tomography (CT) scan and magnetic resonance imaging (MRI) varied over time. To exclude primary metastases, a chest X-ray and a 99mTc-methylene-diphosphonate bone scan were conducted, since 1991 a CT scan of the chest was mandatory as well. Follow-up analyses included X-rays of the chest and primary tumor site in intervals defined by the appropriate COSS protocol. In case of systemic metastases at any time after initial diagnosis, a complete restaging was performed.

### 2.3. Treatment

Treatment including preoperative (neoadjuvant) and postoperative chemotherapy and surgery was to be performed according to the COSS protocols active at enrolment [10–13, 15–17]. In brief, all protocols included varying combinations of high-dose methotrexate with leucovorin rescue, doxorubicin, cisplatin, and/or ifosfamide and sometimes others.

Local therapy was to be performed by surgery during weeks 9 to 11 of therapy, depending on the employed protocol. The type of resection was decided by the local surgeon but it was recommended to attempt wide or radical resections [18] and, if present, it was also recommended to completely resect all primary metastases [17].

### 2.4. Data Collection and Definition of Variables

All variables were collected prospectively and evaluated for distribution within the evaluated patient cohort and for possible correlations with outcome.

*Patient age and sex*: Self-explanatory.

*Tumor site*: Tumor site within the foot was classified by us into one of the three anatomic parts of the foot (phalanges, metatarsal bones, and tarsus) according to the specific bone involved.

*Tumor size*: Absolute tumor volume as measured by initial imaging.

*Primary metastases*: Primary systemic dissemination was assumed whenever metastases other than skip lesions were detected on initial staging, except when the suspicion was later excluded by surgery with negative histology. Patients with a radiologic diagnosis of primary metastases who never underwent surgery for the suspected metastases were included among those with primary dissemination.

*Alkaline phosphatase (AP) and lactate dehydrogenase (LDH)*: Serum levels of AP and LDH were obtained at initial diagnosis. Levels were considered as elevated (E) if they exceeded the upper limit of normal (N) as stated by the local laboratory.

*Symptoms and their duration*: Most COSS protocols, except for those active between 1985 and 1990, included an assessment of symptom duration. The interval between the onset of pain and/or tumor-associated swelling and biopsy/primary operation was counted in days.

*Delay of chemotherapy*: The lag time from diagnostic procedure to the first day of chemotherapy. A treatment delay was arbitrarily defined as an interval of longer than 21 days.

*Timing of surgery*: Primary surgery was assumed whenever an attempt to remove the primary lesion had been performed before the initiation of chemotherapy, whether this had been done with or without the knowledge of the correct diagnosis, whereas primary chemotherapy was assumed if the start of chemotherapy had preceded surgery.

*Type of local surgery*: The surgical procedures were divided into amputation and foot-saving resections as final solution.

*Complete surgical remission (CR)*: A complete surgical remission was assumed only when all detectable tumor foci were removed during first-line therapy. If no complete surgical remission could be achieved, the day after diagnostic biopsy was considered the day of the first event.

*Tumor response*: Response to preoperative chemotherapy was assessed histologically according to the six-grade scale of Salzer-Kuntschik et al. A good response was defined as less than 10% viable tumor residues (response grades 1–3), poor tumor response in case of more than 10% vital tumor cells (grade 4–6) [19].

### 2.5. Statistical Methods

All eligible patients were evaluated on an intent-to-treat basis. All parameters were investigated by univariate techniques. The Kaplan–Meier method [20] was used for survival analysis, and for analysis of the subgroups according to the defined variables, the log-rank test (Mantel-Cox test) or, if appropriate, Breslow's test (generalized Wilcoxon test) was used for comparisons [21–23]. Overall survival was calculated from the time of diagnostic biopsy until death. Event-free survival was calculated until death or first event, whatever occurred first. Patients who never achieved a complete surgical remission were assumed to have suffered an event on day one after diagnostic biopsy.

All *P* values are two-sided, and significant implies *P* < 0.05. SPSS version 22.0 (SPSS Inc., Chicago, IL) was used for statistical calculations.

## 3. Results

We identified 30 patients registered as having osteosarcomas of the foot within the COSS database. Seven of these were excluded from further analyses: five low-grade osteosarcomas (three low-grade central and two parosteal), one osteosarcoma occurred as a secondary malignancy (following B-cell lymphoma), and one benign bone lesion originally misdiagnosed as osteosarcoma, leaving 23 patients with primary high-grade osteosarcomas for statistical analyses (Table 1). The diagnosis of osteosarcoma was made or confirmed by a member of the COSS reference pathology panel in 19 of 23 eligible patients, while four samples were seen by local pathologists only. Patients were registered by 18 institutions from three different European countries (Germany 14, Austria 3, and Switzerland 1).

There were 13 males and ten females, and median age was 32 years (range: 6–58 years). Among 21 of the 23 patients with information on prediagnostic symptoms, eight (38%) complained of pain only, two (10%) registered swelling only, and eleven (52%) reported both, resulting in a total of 19 (90%) patients with pain and 13 (62%) with swelling. In 20 of the 23 patients with relevant data available, the median duration between first symptoms and diagnostic biopsy was 154 days (range: 21–1940 days). The patient with the longest prediagnostic interval had received multiple previous biopsies, with diagnoses ranging from bone cyst to fibrous dysplasia, prior to the diagnosis of osteosarcoma.

Localization of the primary tumor was as follows: in two patients a phalanx (9%), in five patients a metatarsal bone (21%), and in 16 patients a tarsal bone (70%). Absolute tumor volume was documented for 10 of the 23 patients, the median being 31.5 cm^3^ (range: 3–54 cm^3^). All tumors were T1 tumors (<8 cm) according to AJCC staging system (Table 1). Three patients had evidence of primary metastases: one had ipsilateral inguinal lymph node involvement and two suffered from pulmonary metastases.

Among 20 of the 23 patients with appropriate information, serum alkaline phosphatase (AP) levels at diagnosis were normal in 15 (75%) and elevated in five (25%). Among 19 out of 23 patients with available information on lactate dehydrogenase (LDH) serum levels at diagnosis, these were normal in 16 (84%) and elevated in three (16%).

Eighteen of the 23 patients received preoperative chemotherapy, while five had primary surgery (three prior to receiving the correct diagnosis and two thereafter). The median duration between diagnostic biopsy/primary surgery and start of chemotherapy was 28 days (range: 1–83 days).

Twenty-one of the 23 patients (87%) achieved a macroscopically complete surgical remission of all tumor sites (Tables 1 and 2). The remaining two were not operated for pulmonary metastases, one of these had progression of primary metastases and the other developed metastases during preoperative chemotherapy. Among the 23 patients with known surgery of their primary tumor, 19 (83%) received only one surgical procedure until obtaining their best total surgical outcome and 4 (17%) received two surgical procedure (three patients received amputation after incomplete primary resection and one patient received complete resection of pulmonary metastases). In total, 19 patients (83%) underwent amputations and four (17%) foot-saving resections (Tables 1 and 3). Among these four patients, three received a resection with wide margins and one with marginal margins. The patient receiving resection with marginal margins had primary pulmonary metastases, which were not operated, and developed a large local recurrence.

Four of 17 (25%) tumors which were resected following preoperative chemotherapy and for whom information on histological response was available responded well to preoperative chemotherapy (<10% viable tumor), and thirteen (75%) responded poorly (Tables 1 and 4).

Twenty-two patients received systemic chemotherapy for their primary disease; information on the drugs used was available for 21. Among these, all 21 received doxorubicin, 21 received cisplatin (100%) (two additional carboplatin), 19 ifosfamide (90%), 16 high-dose methotrexate (76%), and five etoposide (24%) (Table 1).

**Figure 1:**
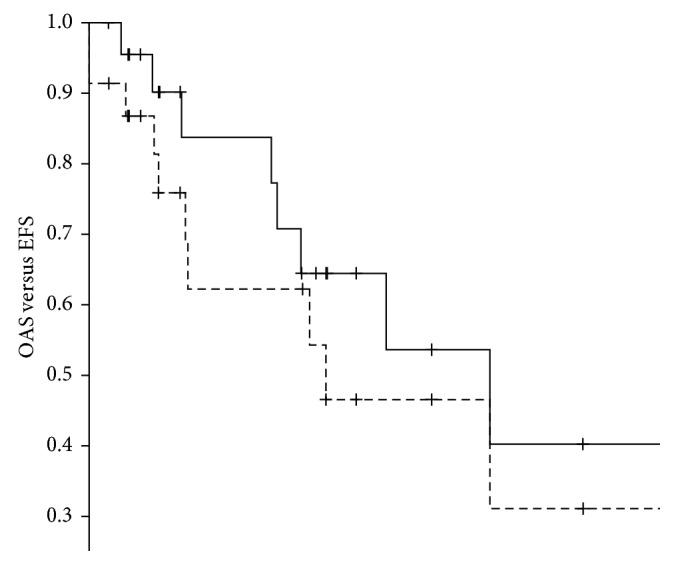
Overall survival (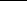) (95% confidence interval: 0.0–10.8) and event-free survival (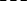) (95% confidence interval: 3.6–14.3) of the 23 patients with high-grade osteosarcoma of the foot.

After a median follow-up of 4.2 years (range: 0.4–18.5 years) for all 23 patients and 4.8 years (range: 0.4–18.45 years) for the 15 survivors, three- and five-year survival estimates were 84% (standard error (SE) 8.6%) and 64% (SE 12%), respectively (Figure 1). Among the 15 survivors, thirteen were in first complete remission, one was lost to follow-up while in first recurrence, and another one was alive with his third recurrence. Of the eight patients who died, six suffered from progressive disease (two without ever having achieved a complete remission, one in first, one in second, and two after third recurrence), one of a secondary malignancy (Ewing sarcoma), and one of an unknown cause during first recurrence (Tables 1 and 5).

Among 21 patients in whom complete surgical remission was achieved, thirteen remained event free and eight experienced an event. Among these, five developed lung metastases (de novo, 1 following complete removal of primary lung metastases), one de novo ipsilateral inguinal lymph node metastases, and one a recurrence in the ipsilateral proximal lower leg following complete surgery of both the primary tumor and primary (inguinal) lymph node metastases. In addition, one patient died from a secondary malignancy (Ewing sarcoma) (Table 1). Three- and five-year event-free survival estimates were 62% (SE 12%) and 54% (SE 13%), respectively. There was no significant difference in overall and event-free survival between the first 18 years of patient recruitment and the second 18 years.

Event-free survival (EFS) and overall survival (OAS) correlated with primary metastatic status and best surgical remission status (Table 6).

## 4. Discussion

Osteosarcoma of the foot is exceedingly rare, and consequently the available information on patient and tumor characteristics, optimal management, and outcome is very limited. Therefore, we decided to investigate the greatest time span possible (36 years) using the data prospectively collected by the Cooperative German-Austrian-Swiss Osteosarcoma Study Group. We were able to analyze 23 eligible patients with primary high-grade osteosarcoma of the foot, which represents one of the largest cohorts of such patients reported to date.

While recommended diagnostic and therapeutic procedures have varied to some extent during this prolonged period, the overall results of osteosarcoma therapy have not [24–27], so we believe our findings hold true even for today. Low-grade as well as secondary osteosarcomas were excluded, as their biology and/or treatment differs from the more common primary high-grade central osteosarcomas.

Concerning patient-related variables, we observed the same slight male predominance as known for extremity osteosarcoma [28], but, similar to others [1, 2], a considerably older median age of 32 years. As in osteosarcoma, in general [13], pain was the most frequent presenting symptom. The median duration between first symptoms and diagnostic biopsy was 154 days (range: 21–1940 days), which is shorter than that described in other series of osteosarcomas of the feet [2, 4] but longer than that we have observed for other extremity osteosarcomas (median: 69 days) [13].

Like others [1, 5], we observed the tarsal bones to be the most frequently affected site within the foot. In our 23 patient cohort, three had evidence of primary metastases upon imaging, comparable to the situation in osteosarcoma of other sites [13, 29]. Two had lung metastases and one had lymph node metastases, the latter being rather unusual for osteosarcoma [30]. Compared to osteosarcoma in general [31], fewer patients from our series presented with elevated alkaline phosphatase levels, probably correlating with their smaller tumor volumes, while the rate of elevated lactate dehydrogenase was similar [32].

Our patients with osteosarcoma of the foot received the same multimodal therapy including chemotherapy and surgery as patients with osteosarcoma in general. While the more frequent osteosarcomas of long extremity bones have witnessed a major shift from amputation towards limb-saving surgery over the past several decades [14], we did not observe such a trend in this series, where three quarters of all affected feet were either completely or partially amputated.

Compared to osteosarcoma in general, where approximately half of all tumors respond well to preoperative chemotherapy [13, 28, 33], only one quarter of 16 evaluable pedal osteosarcomas from our series did so. We were not able to extract information regarding response from other published series, so that this disparity must probably be considered a novel finding for which there is no immediate explanation besides the small cohort size. The biology underlying this apparent difference remains to be elucidated.

Like in extremity osteosarcoma in general [28], most patients from our series achieved a first complete surgical remission. The recurrence rate and the time to recurrence were also similar to that which our group has observed for extremity osteosarcoma in general [13, 34]. Interestingly, there were no local recurrences as first event, a result which may have been favored by the aggressive, mostly ablative surgical approach employed. Given that wide margins may be difficult to achieve by foot-salvaging procedures, margins correlate with the local recurrence risk [35], prognosis following local osteosarcoma recurrence is very poor [36] and gait performance is often quite good following partial or even complete amputation of the foot [37]; we believe that such an aggressive surgical approach is well justified.

The recurrences we observed were mostly lung metastases, again as well known for osteosarcoma in general [28, 34]. Two patients had either primary or secondary lymph node involvement, which is rather unusual for this particular malignancy [30]. However, the small numbers prohibit making definitive statements about whether the risk for lymphatic spread is truly higher than for osteosarcomas of other sites. Metastases in other published series were usually pulmonary [1]. Nevertheless, we would recommend careful assessment of the ipsilateral lymphatic drainage as part of staging and follow-up of patients with an osteosarcoma of the foot.

Given the very similar recurrence rates already discussed above, it comes as no surprise that the 5-year event-free and overall survival rates are also similar to those observed in other series which included both localized and primary metastatic osteosarcomas [14, 26, 38]. Even though tumor size is a very well-established prognostic factor and osteosarcomas of the foot are more likely to be detected at smaller size, the obtained results are certainly not superior to those our group has achieved in other long-bone extremity osteosarcomas [13]. We can only assume that this may be due to a somewhat different tumor biology which also manifests in the low response rate to preoperative chemotherapy. As a note of caution, some papers on foot osteosarcomas have reported higher cure rates, albeit based upon even smaller patient numbers [4].

Patients with primary metastases are known to be associated with inferior event-free and overall survival rates [13, 30, 39, 40], which was also seen in our cohort, where none of the three patients with primary metastases survived. Complete surgical remission, mostly achieved by amputation, was the strongest positive predictive factor for EFS and OS in our cohort. In this context, we have to emphasize that the subgroup of patients not receiving complete surgical remission consisted of only 2 of the 23 patients and these patients had inoperable primary metastases, respectively, progressive disease under chemotherapy. Nonetheless, our finding is in accordance with the general osteosarcoma literature [13, 28]. When investigating other factors for potential correlations with prognosis, such as tumor site within the foot, size, elevated serum LDH or AP levels, or response to preoperative chemotherapy [1, 13, 32, 41–43], we did not observe significant correlations with either event-free or overall survival, which may of course have been due to the limited number of patients included in our study.

## 5. Conclusion

Our study is one of the largest cohorts of patients with osteosarcoma of the foot reported to date despite the relatively small collection of only 23 patients. Using the same treatment strategy as employed in extremity osteosarcomas in general, we also achieved similar results. Primary metastatic status and surgical outcome correlated with prognosis. These results argue in favor of treating osteosarcomas of the foot like other extremity osteosarcomas and further highlight the importance of achieving complete surgical remission, especially regarding the poor response of the tumors to neoadjuvant chemotherapy.

## Figures and Tables

**Table 1:** Patient and tumor characteristics, treatment, and outcome.

Pat. no.	Age	Sex	Tumor site	Tumor size (ml)	Primary mets.	AP	LDH	Pre-op chemotherapy	Surgical remission	Type of surgery	Tumor response	Postoperative chemotherapy	Event	Further therapy	EFS (years)	OAS (years)	Status
1	10	F	Tarsus (calcaneus)	40.56 max.; dimension: 6.5 cm	None	N	N	A, M, I, P	CR	Amputation lower leg below knee	Poor	A, M, I, E	SMD (Ewing sarcoma)		8.9	8.9	DOC-SMD
2	12	F	Tarsus (os cuneiforme lateral)	5.5 max.; dimension 2.5 cm	None	N	N	A, M, I, P	CR	Amputation forefoot in Chopart joint	Good	A, I, P	None		13	13	LFU-CR1
3	12	M	Tarsus (talus: position in the extensor aspect)	12.08 max.; dimension 3 cm	None	—	—	A, M, I, P	CR	Resection of talus, half os naviculare, and distal part of lateral calcaneus incl. sinus tarsi; reimplantation of proximal talus: tumor in 1 cm distance	Poor	A, M, I, P	None		11	11	LFU-CR1
4	13	M	Tarsus (talus medial part)	31.2 max.; dimension 5 cm	2–5 lung	E	E	A, M, I, P	No	En bloc resection distal part of tibia, fibula and talus	Poor	A, P, E, HD-I	No CR progressive lung mets., local rec.	n.f.s.	0	0.7	DOD-primary disease
5	13	M	Tarsus (calcaneus)	36.11 max.; dimension 6.5 cm	None	E	N	A, M, I, P	CR	Amputation of foot: exarticulation upper ankle joint	Poor	A, M, I, P	None		7.6	7.6	LFU-CR1
6	13	M	Tarsus (calcaneus)	54 max.; dimension 8 cm	None	E	N	A, M, P	CR	Amputation lower leg below knee	Poor	A, M, P	None		0.86	0.86	CR1
7	23	M	Tarsus (calcaneus)	—	None	N	N	A, M, P	CR	Amputation lower leg below knee	Poor	A, M, P	None		0.9	0.9	LFU-CR1
8	25	F	Tarsus (calcaneus)	—	None	N	N	COSS 96, n.f.s	CR	Amputation lower leg below knee	—	n.f.s.	None		4.8	4.8	LFU-CR1
9	25	F	Tarsus (calcaneus)	—	None	N	N	A, M, I, P	CR	First operation: excochleation of cyst; second operation: amputation of lower leg below knee	Poor	A, M, I, P	Lymph node metastases ipsilateral groin	Second- and third-line chemotherapy	0.8	1.4	DOD-Rec1
10	29	F	Tarsus (calcaneus)	—	None	E	N	A, M, P	No	Amputation lower leg n.f.s	Poor	A, M, I, P, E	No CR: pulmonary filiae during preoperative chemo, mets. HWK4 and BWK 3	Corporectomy of HWK4 and BWK3, mets. Os pubis, fourth rib	0	2.1	DOD-primary disease
11	32	M	Tarsus (os naviculare + cuneiforme III)	—	Lymph node; left groin	N	E	A, M, I, P	CR	Amputation: foot through calcaneus; resection of groin metastases	Poor	A, M, I, P	Rec. lower leg and lung mets.	Resection of distal fibula	2.2	4.2	DOD-Rec2
12	33	F	Tarsus (os naviculare)	—	None	E	E	A, M, I, P	CR	Amputation of forefoot	Good	A, I, P, E, M	Lung mets.	High-dose chemo rejected	4.9	5.0	LFU-Rec1
13	38	M	Tarsus (calcaneus)	32 (5 × 5 × 2.5 cm)	None	N	N	A, M, I, P	CR	First operation: intralesional excochleation of a “cyst,” second operation: amputation lower leg below knee	N.A. (primary OP)	A, M, I, P	Lung mets. 3x	2 × metastasectomies	2.2	4.1	DOD-Rec3
14	38	F	Tarsus (calcaneus)	51 max.; dimension 7 cm	None	N	—	A, M, I, P	CR	Amputation lower leg below knee	Poor	A, M, I, P	None		1.1	1.1	LFU-CR1
15	56	M	Tarsus (talus)	—	None	N	N	A, P, I	CR	Amputation distal lower leg	Good	A, P, I	None		2.0	2.0	LFU-CR1
16	58	M	Tarsus (calcaneus)	—	None	N	N	—	CR	First operation: resection of calcaneal cyst: second operation: amputation lower leg below knee	N.A. (primary OP)	A, P, I	None		0.4	0.4	LFU-CR1
17	11	F	Metatarsale IV: proximal right part	—	None	N	N	A, P, M	CR	Resection ray IV, metatarsale III and V reconstruction of axis III with fibula	Poor	A, P, M, E, I	None		4.0	4.0	LFU-CR1
18	39	F	Metatarsale IV: mediocranial	3.31 max.; dimension 3.5 cm	None	—	—	—	CR	Partial resection of ray III–V through cuboid and cuneiforme III	N.A. (primary OP)	A, I, P, M	Lung mets.	Rec. I–III: 3 × wedge resection of affected pulmonary site	5.3	18.45	CR3
19	44	M	Metatarsale II, os cuneiforme II and III	—	None	N	N	A, I, P	CR	Amputation forefoot in Chopart joint	Poor	A, P, I	None		1.6	1.6	CR1
20	44	M	Metatarsale V	—	Lung	N	N	A, P, I	CR	First operation: amputation atypical in Chopart joint; second operation: wedge resection left lower lobe	Good	I, P, M, A	Lung mets. bilateral	No further therapy: patient will	1.5	4.72	DUC-Rec1
21	45	M	Metatarsale I	—	None	N	N	A, P, I	CR	Amputation ray I and II and os naviculare	Poor	A, P, I	None		2.0	2.0	LFU-CR1
22	45	F	Phalanx I: proximal part	—	None	—	—	None; patient disbelieved diagnosis	CR	Amputation ray I and II right	N.A. (primary OP)	None	Rec. 1: lung mets., Rec. 2: lung + popliteal fossa, and Rec. 3: lymph node mets.: lung, mediastinal, and abdominal	2x wedge resection, amputation right leg	1.5	6.6	DOD-Rec3
23	57	M	Phalanx distalis I	31 max.; dimension 5 cm	None	N	N	—	CR	Amputation phalanx I	N.A. (primary OP)	A, P, I	None		1.6	1.6	LFU-CR1

Pat. = patient; No. = number; status = status at the last available follow-up; F = female; M = male; N = normal; E = elevated; N = normal; A = doxorubicin; M = methotrexate; I = ifosfamide; HD = high dose; P = cisplatin; E = etoposide; CR = complete surgical remission (primary tumor and metastases); good/poor tumor response = </≥10% viable tumor following pre-op. chemotherapy; SMD = secondary malignant disease; n.f.s. = not further specified; mets. = metastases; OP = operation; N.A. = not applicable; DOC = death of other cause; LFU = lost to follow-up; DOD = death of disease; Rec. = recurrence.

**Table 2:** Complete surgical remission in localized and metastatic disease.

Surgical remission	Number of patients with localized disease	Number of patients with metastatic disease	Number of all patients
Number of patients	20	3	23
Complete resection of primary tumor	20	3	23
Complete resection of metastases	0^∗^	2	2
Complete surgical remission	19	2	21

One patient with localized disease developed pulmonary metastases during preoperative chemotherapy and did not receive metastasectomy because of progressive disease.

**Table 3:** Type of surgery.

Type of surgery	Number of patients with localized disease	Number of patients with metastatic disease	Number of all patients
Amputation	17	2	19
Resection	3	1	4

**Table 4:** Tumor response to preoperative chemotherapy.

Tumor response	Number of patients with localized disease	Number of patients with metastatic disease	Number of all patients
Good (less than 10% viable tumor cells)	3	1	4
Poor (more than 10% viable tumor cells)	11	2	13
Not applicable (primary surgery)	5	0	5
Not documented	1	0	1

**Table 5:** Outcome at the last follow-up.

Outcome	Number of patients with localized disease	Number of patients with metastatic disease	Number of all patients
Died	5	3	8
Alive	15	0	15
Alive CR1	13	0	13
Alive Rec1-LFU	1	0	1
Alive CR3	1	0	1

CR = complete surgical remission (primary tumor and metastases); CR1 = first complete surgical remission; CR3 = third complete surgical remission; Rec. 1 = first recurrence; LFU = lost to follow-up.

**Table 6:** Univariate analysis of overall and event-free survival.

Survival	Number of patients	Percent	Event-free survival			Overall survival		
Variable			5 year (%)	SE (%)	*P* (log-rank)	5 year (%)	SE (%)	*P* (log-rank)
Total	23							
Age								
<32 years (median)	12	52	64	15	0.438	61	15	0.828
>32 years	11	48	39	21		67	20	
Sex								
Male	13	57	44	18	0.791	39	17.5	0.235
Female	10	43	63	18		89	10.5	
Tumor site								
Tarsus	16	70	49	16	0.903	57	15	0.503
Other	7	30	71	17		80	18	
Tumor size								
<31.5 cm^3^ (median)	5	50	80	18	0.786	80	18	0.366
>31.5 cm^3^	5	50	67	27		67	27	
Primary metastases								
No	20	87	66	13	0.005	78	11	0.008
Yes	3	13	0	0		0	0	
AP								
Normal	15	75	60	16	0.216	57	16	0.568
Elevated	5	25	30	24		53	25	
LDH								
Normal	16	84	68	14	0.076	62	15	0.291
Elevated	3	16	0	0		33	27	
Duration of symptoms								
<154 days (median)	11	55	76	16	0.188	76	16	0.463
>154 days	9	45	29	22		51	20	
Delay of chemotherapy								
<21 days	8	35	58	19	0.532	73	17	0.441
>21 days	15	65	52	17		57	17	
Timing of operation								
After preoperative chemotherapy	18	78	52	14	0.929	64	13	0.587
Primary surgery	5	22	67	27		67	27	
Type of surgery								
Resection	4	17	75	22	0.660	75	22	0.347
Amputation	19	83	45	15		60	14	
Surgical remission								
Complete remission	21	95	60	13	0.000	72	12	0.000
Macroscopic residual	2	9	0	0		0	0	
Tumor response								
Good	4	24	38	29	0.980	67	27	0.435
Poor	13	76	64	15		59	16	

5-year event-free and overall survival and *P* values in the log-rank test for all variables (see Data collection and Definition of Variables). SE = standard error; *P* = two-sided *P* values.
